# Machine learning data sources in pediatric sleep research: assessing racial/ethnic differences in electronic health record–based clinical notes prior to model training

**DOI:** 10.3389/frsle.2024.1271167

**Published:** 2024-02-14

**Authors:** Mattina A. Davenport, Joseph W. Sirrianni, Deena J. Chisolm

**Affiliations:** ^1^Abigail Wexner Research Institute, Center for Child Health Equity and Outcomes Research, Nationwide Children's Hospital, Columbus, OH, United States; ^2^Department of Pediatrics, College of Medicine, The Ohio State University, Columbus, OH, United States; ^3^Abigail Wexner Research Institute, IT Research and Innovation, Nationwide Children's Hospital, Columbus, OH, United States

**Keywords:** sleep, equity, public health, primary care, informatics, machine learning, population health

## Abstract

**Introduction:**

Pediatric sleep problems can be detected across racial/ethnic subpopulations in primary care settings. However, the electronic health record (EHR) data documentation that describes patients' sleep problems may be inherently biased due to both historical biases and informed presence. This study assessed racial/ethnic differences in natural language processing (NLP) training data (e.g., pediatric sleep-related keywords in primary care clinical notes) prior to model training.

**Methods:**

We used a predefined keyword features set containing 178 Peds B-SATED keywords. We then queried all the clinical notes from patients seen in pediatric primary care between the ages of 5 and 18 from January 2018 to December 2021. A least absolute shrinkage and selection operator (LASSO) regression model was used to investigate whether there were racial/ethnic differences in the documentation of Peds B-SATED keywords. Then, mixed-effects logistic regression was used to determine whether the odds of the presence of global Peds B-SATED dimensions also differed across racial/ethnic subpopulations.

**Results:**

Using both LASSO and multilevel modeling approaches, the current study found that there were racial/ethnic differences in providers' documentation of Peds B-SATED keywords and global dimensions. In addition, the most frequently documented Peds B-SATED keyword rankings qualitatively differed across racial/ethnic subpopulations.

**Conclusion:**

This study revealed providers' differential patterns of documenting Peds B-SATED keywords and global dimensions that may account for the under-detection of pediatric sleep problems among racial/ethnic subpopulations. In research, these findings have important implications for the equitable clinical documentation of sleep problems in pediatric primary care settings and extend prior retrospective work in pediatric sleep specialty settings.

## 1 Introduction

Racial/ethnic disparities are well documented and persistent in pediatric sleep at the population level (Billings et al., 2021; Meltzer et al., 2021; Reynolds et al., 2023). However, these disparities are often preventable and linked to social determinants of health at the individual, family, healthcare, and broader community/societal levels (Billings et al., 2021; Fanta et al., 2021; Yip et al., 2022; Clarkson-Townsend et al., 2023; Gueye-Ndiaye et al., 2023). Pediatric primary care is ideal for preventing pediatric sleep disparities at the population level, yet providers in this setting typically lack the time and resources necessary to identify sleep problems (Honaker and Saunders, 2018; Mosher and Piccinini-Vallis, 2022; Williamson et al., 2022; Golden et al., 2023). Efficient machine learning and clinical decision support tools embedded in the pediatric primary care electronic health record (EHR) are needed to support universal screening of pediatric sleep problems at the population level (Anan et al., 2023). In addition, EHR-embedded machine learning tools for data collection are essential to include patient-self report and aid providers with limited personnel and time constraints in pediatric primary care (Honaker et al., 2019; Huffstetler et al., 2022; Willis et al., 2022). Therefore, EHR-embedded machine learning tools have the potential to innovatively address modifiable pediatric sleep care gaps (Kang et al., 2021; Ramgopal et al., 2023).

Despite their promise, EHR-embedded machine learning tools also have the capability of worsening racial/ethnic disparities due to inherently biased healthcare data sources used for training machine learning models (Chen et al., 2023). In the context of the EHR, training data commonly used for machine learning in pediatric sleep may be inherently biased for two reasons: historical biases and informed presence (Hamilton et al., 2021). Historical biases include an overrepresentation of non-Hispanic white patients in epidemiologic pediatric sleep cohorts, which have commonly leveraged patients with confirmed sleep diagnoses (Meltzer et al., 2010; Honaker and Meltzer, 2016). Informed presence occurs when patients navigate the healthcare system but experience barriers that yield variable interactions across racial/ethnic subpopulations (Phelan et al., 2017). Informed presence is critical to account for when developing EHR-embedded machine learning tools because it can have downstream effects on how racial/ethnic subpopulations are classified, measured, and/or represented in healthcare data sources (Phelan et al., 2017). Therefore, researchers should assess for such biases (e.g., historical or induced by patients' healthcare navigation) that may be inherent in healthcare data sources prior to training and developing models for automated solutions (Huang et al., 2022).

As posited in the Peds B-SATED framework by Meltzer et al. (2021), pediatric sleep problems can be multidimensional and include unhealthy sleep behaviors (B), poor sleep satisfaction (S), difficulty with alertness during waking hours (A), inappropriate sleep timing (T), low sleep efficiency (E), and inadequate sleep durations for age (D). A reliance on sleep diagnoses and polysomnography data limits epidemiologic and population pediatric sleep research by failing to capture all the subclinical characteristics described in the Peds B-SATED framework (Yang et al., 2023). Leveraging clinical note data from the EHR is a way to improve our identification of Peds B-SATED in primary care settings using EHR-embedded machine learning tools. Natural language processing (NLP), a machine learning model for understanding language and contextualized nuances in EHR free-text clinical notes, is an innovative and available approach for capturing Peds B-SATED framework subclinical characteristics (Gianfrancesco and Goldstein, 2021; Rahman et al., 2022). However, this data source and approach are not exempt from being inherently biased due to the reliance on clinical notes, telephone notes, patient–provider messages, and other text-based fields that are shaped by informed presence in healthcare systems (Rozier et al., 2022; Walk et al., 2022). Recent NLP work used to audit clinical notes has found that the language used to describe providers' recognition of patients' reported characteristics (e.g., sociodemographic and clinical) varies by race/ethnicity (Thompson et al., 2021; Sun et al., 2022). Therefore, it is important to assess how Peds B-SATED keywords and global dimensions are documented and captured across racial/ethnic subpopulations in NLP training data sources prior to model training and using these methods to develop EHR-embedded machine learning tools.

To address this knowledge gap, utilizing clinical notes from patients seen in pediatric primary care, the current study included two objectives: (1) A least absolute shrinkage and selection operator (LASSO)–normalized logistic regression model was used to investigate whether there were racial/ethnic differences in documentation of Peds B-SATED keywords. (2) A mixed-effects logistic regression was used to determine whether the odds for the presence of global Peds B-SATED dimensions also differed across racial/ethnic subpopulations. We hypothesized that racial/ethnic differences in the documention of keywords and global dimensions would be observed by LASSO regression and multilevel modeling approaches.

## 2 Methods

### 2.1 Participants

We conducted a cross-sectional cohort study of 44,244 patients, 5 to 18 years old, seen in a pediatric primary care network at a large academic medical center. We excluded infants and early childhood youth due to developmental reliance on caregivers to support sleep, which would require an extensive and separate set of pediatric behavioral sleep medicine keywords and phrases. The protocol was approved by the institutional review board at Nationwide Children's Hospital.

### 2.2 Data source and procedures

#### 2.2.1 DeepSuggest clinical note search engine

DeepSuggest is an internally developed and validated clinical note search engine at Nationwide Children's Hospital (Moosavinasab et al., 2021). DeepSuggest queries clinical notes by a set of keywords and filters by note type, provider type, department specialty, date range, age range, and patient information such as name, date of birth, and medical record number. In addition to facilitating a search of EHR-based clinical notes, DeepSuggest expands query terms by recommending related or similar search keywords based on the similarity of keyword Word2Vec embeddings calculated across all notes in the repository on the backend (Mikolov et al., 2013). During this process, duplicate notes are not included.

We utilized DeepSuggest to expand our initial keywords and retrieve clinical notes that contained at least one Peds B-SATED keyword or phrase. For vocabulary expansion, we entered our initial set of Peds B-SATED keywords into DeepSuggest, and it determined recommended keywords based on their relevance. This vocabulary expansion increased our keywords by including those with common misspellings (e.g., “insomia”), inconsistent punctuation (e.g., “sleepwalking” vs. “sleep-walking”), abbreviations, and synonyms (e.g., “difficulty staying awake during the day” vs. “sleepy during the day”).

#### 2.2.2 Predefined keyword features set approach

We desired to cluster clinical notes into groupings based on their presence of global Peds B-SATED dimensions, so we applied a predefined keyword features set approach. This is an NLP approach that uses the presence of each of the keywords as a representation of the clinical note, rather than a predictive NLP model. To convert these Peds B-SATED keyword occurrences into a numerical representation, we searched each clinical note for an occurrence of each of the 178 keywords, using case-invariant matching, and phrases, using regular expressions. If a keyword or phrase was found, we would mark the keyword's corresponding index in a 178-dimensional vector with a 1; if no occurrence of that keyword was found, its value would be 0. At the end of this process, each note had a corresponding 178-dimensional binary keyword vector. In the end, using a predefined keyword features set containing 178 Peds B-SATED keywords, we queried all the clinical notes for patients between the ages of 5 and 18 from January 2018 to December 2021.

### 2.3 Analyses

#### 2.3.1 LASSO regression model

To investigate whether there were racial/ethnic differences in the documentation of Peds B-SATED keywords, we fit a LASSO regression model predicting patients' race/ethnicity using the occurrence of Peds B-SATED keywords as the input feature. This model assessed the Peds B-SATED keywords used in a patient's clinical note(s) to detect if they differed across race/ethnicity subpopulations in our cohort.

For this LASSO regression model, we performed two analytic steps. The first step was focused on the overall predictiveness of the Peds B-SATED keywords. We trained a LASSO regression model using 10-fold cross-validation. This included using the combined holdout subsets of data from each fold to evaluate the model's overall performance parameters: area under the receiver operating characteristic curve (AUC ROC), precision, recall, accuracy, and F1-scores. To ensure that our results were rigorous and meaningful, we repeated our 10-fold cross-validation training 1,000 times using bootstrapping to randomly resample the data in the training folds at each step, stratified by label (ensuring the racial/ethnic proportion of the training data remains constant). This analysis investigated the predictive capability of the Peds B-SATED keywords.

The second step was focused on identifying which Peds B-SATED keywords were most influential for predicting each racial/ethnic patient subpopulation. In this step, we trained another LASSO regression model using all of the data simultaneously. By examining the coefficients of each Peds B-SATED keyword, we reported the 10 most common keywords used for each racial/ethnic subpopulation. This step also included predicting patients' race/ethnicity from the Peds B-SATED keywords in their clinical notes. To do this, we collapsed patient race/ethnicity into six categories: non-Hispanic Black, Hispanic/Latino, non-Hispanic white, non-Hispanic Asian, non-Hispanic Multiracial (more than two races), and Other Race/Ethnicity (including non-Hispanic Native Hawaiian or Other Pacific Islander, non-Hispanic American Indian or Alaska Native, and no information given/unknown/refuses to answer). Race is collected by patient report at the time of registration and entered into Epic by registration staff. Registration staff are trained to directly ask the caregiver/patient to select the race/ethnicity category they most identify with and have the option to not report this information. For predicting patients' race/ethnicity, we aggregated each patient's Peds B-SATED keywords vector for all of their clinical notes and then used that in the LASSO regression model as the input features to predict patients' race/ethnicity category.

#### 2.3.2 Mixed-effects logistic regression

Descriptive analyses were used to count the incidence of patients that had global Peds B-SATED dimensions present. The percentages of patients with global Peds B-SATED dimensions are reported. Mixed-effects logistic regression models (e.g., clinical notes nested within patients, with a random effect for patients) were used to predict whether the presence of global Peds B-SATED dimensions differed by patients' race/ethnicity. Statistical models were adjusted for covariates at both the note and patient levels. Note-level covariates included clinical note author type (nurse practitioner, physician, integrated pediatric psychologists, social workers, and others/trainees) and note date pandemic status was dichotomized (notes prior to 1 March 2020; notes after 1 March 2020). Patient-level covariates included the following sociodemographic characteristics: biological sex (female vs. male) and age. Logistic mixed-effects models were fitted using the glmer function from the lme4 package, the performance of the fitted models was compared, and the best model was selected using Akaike's information criterion and Bayesian information criterion. A *p*-value of <0.05 was considered statistically significant. Statistical analyses were performed using R version 4.2.2 and R Studio software (Bates et al., 2015).

## 3 Results

### 3.1 Patient cohort characteristics

Our sample consisted of 44,244 patients with 111,078 clinical notes. Of the patients, 51.4% were classified as non-Hispanic Black, 24.6% as non-Hispanic white, 8.1% as non-Hispanic Multiracial, 4.6% as non-Hispanic Asian American, 11.4% as non-Hispanic Other Race/Ethnicity, and15.6% as Hispanic or Latino. The mean age was 11.12 (*SD* = 3.78), and 49.1% were classified as female.

### 3.2 Racial/ethnic differences in documentation of Peds B-SATED keywords

**Figure 1:**
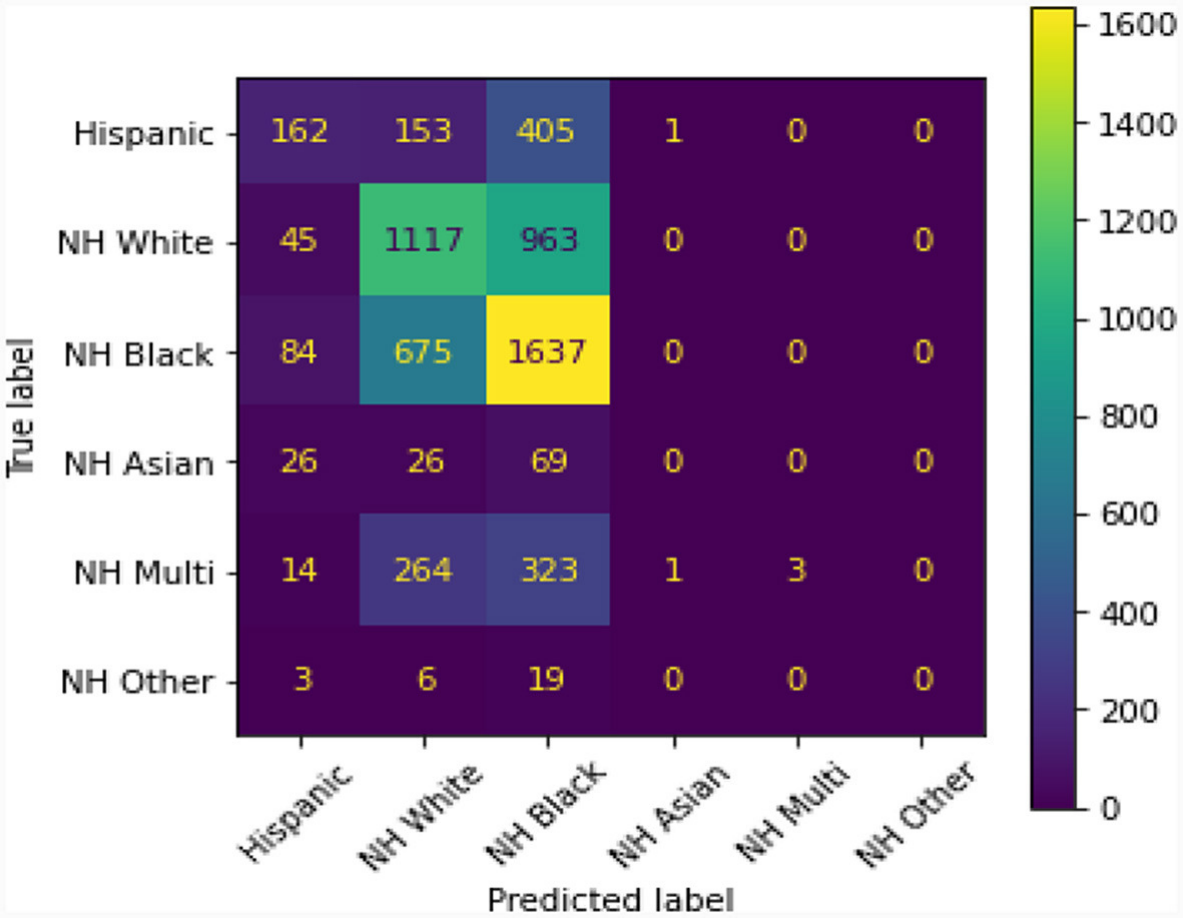
The presented confusion matrix in this figure from the least absolute shrinkage and selection operator regression model revealed that providers' use of Peds B-SATED keywords in clinical notes predicted patients' race/ethnicity. The color spectrum on the right, ranging from 0 to 1,600, displays a near-zero predictive ability in purple and a high predictive ability in yellow. Ideally, the matrix would display that all predicted labels, race/ethnicity, were purple and near 0. NH, non-Hispanic; Multi, Multiracial.

Figure 1 shows a comparison–confusion matrix table. A confusion matrix is a table that is used to display the predictive ability of the LASSO regression model used in this study. Ideally, in this particular case, the predictive ability should be at 0 and not show the model's capability of predicting patients' race/ethnicity from Peds B-SATED keywords. However, this reveals that race/ethnicity across patient subpopulations in our cohort could be predicted by primary care providers' use of keywords in clinical notes and that this difference was most observable among non-Hispanic Black and non-Hispanic white patient subpopulations.

**Figure 2:**
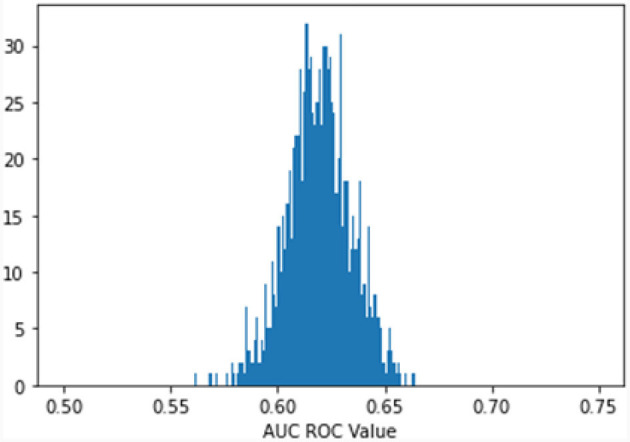
Predict patient race: bootstrap (1,000 reps) 10-fold cross-validation. The Figure displays the distribution of the area under the receiver operating characteristic curve (AUC ROC) for 1,000 bootstrapped iterations of 10-fold cross-validation predicting patient race. AUC ROC is a metric that reflects how well a model can discriminate between categories. A random model will have an AUC ROC score of 0.5, and a perfect model (can separate everything without error) will have a value of 1.0. Our average AUC ROC score is 0.6194, with a standard distribution of 0.0151. In no case was the AUC ROC score at or below 0.5, indicating that the model is always able to discriminate patients' race/ethnicity using the Peds B-SATED keywords from primary care providers' clinical notes.

**Figure 3:**
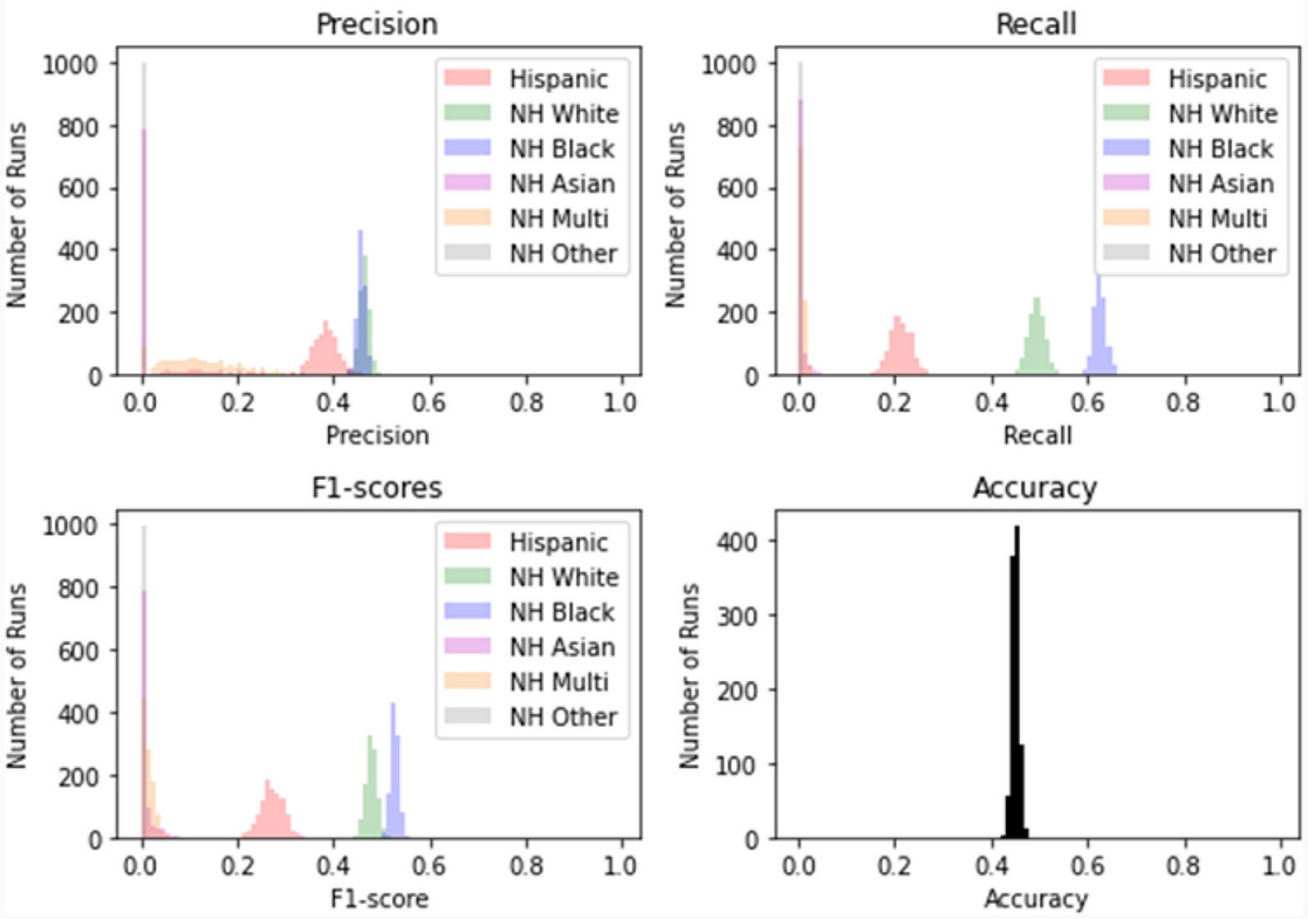
Distribution of scores for 10-fold cross-validation on 1,000 rep bootstrap. The figure displays the specific performance of the least absolute shrinkage and selection operator regression model prediction of racial/ethnic subpopulations across precision, recall, F1-score (the harmonic mean of precision and recall), and the model's overall accuracy. NH, non-Hispanic; Multi, Multiracial.

A model with random classification ability will have an AUC ROC of 0.5, and a perfect model (which can separate everything without error 50% of the time) will have a value of 1.0. Our average AUC ROC score is 0.72. Figure 2 shows in no case was the AUC ROC score at or below 0.5, indicating that this LASSO regression model with Peds B-SATED keywords was always able to learn to discriminate patients by race/ethnicity to some degree, which is an indication of racial/ethnic differences in primary care providers' documentation of Peds B-SATED keywords. Figure 3 shows that non-Hispanic Black patients had the highest overall model F1-score (mean = 0.56) and model recall (mean = 0.68) scores. In addition, non-Hispanic Black patients were similar to non-Hispanic white patients in terms of model precision.

To more specifically demonstrate how providers' documentation of specific Peds B-SATED keywords differed for racial/ethnic subpopulations, Table 1 shows the top 10 Peds B-SATED keywords for each racial/ethnic subpopulation that were documented by pediatric primary care providers, including the coefficient ranking value. The most common Peds B-SATED keyword rankings qualitatively differed across racial/ethnic subpopulations. Most interestingly, non-Hispanic white patients had distinguished Peds B-SATED keywords that may lead to follow-up support and/or a referral to specialty care to address pediatric sleep problems. However, minoritized racial/ethnic subpopulations commonly had Peds B-SATED keywords that encompassed broader descriptions and relevant daytime sequelae of pediatric sleep problems.

**Table 1:** Top 10 Peds B-SATED keywords based on highest ranking coefficients.

**Rank**	**Hispanic**	**NH white**	**NH Black**	**NH Asian**	**NH Multi**	**NH Other**
1	Limb movements (1.112)	Drink caffeine (1.727)	Bedtime schedule (0.943)	Poor focus (0.983)	Difficulty getting to sleep (0.825)	Sleeping difficulties (2.055)
2	Sleep difficulties (1.080)	Anxiety at night (1.501)	Problems with sleeping (0.891)	Up to (num) hours (0.901)	Disruptive behavior (0.560)	Up at night (1.754)
3	Less than (num) hours (0.788)	Limb movements (1.130)	Difficulties Sleep (0.720)	Inadequate sleep (0.794)	Sleeping during the day (0.512)	Cannot sleep (1.189)
4	Sleep (num) hours (0.742)	Night waking (0.912)	Bedwetting (0.563)	Hypersomnia (0.779)	Melatonin (0.492)	Sleep habits (0.621)
5	Misbehaves (0.659)	Impaired sleep (0.809)	Hypersomnolence (0.490)	Difficulties sleeping (0.709)	Sleep disturbances (0.402)	Trouble falling asleep (0.537)
6	Poor sleep pattern (0.634)	Parasomnia (0.721)	Up at night (0.466)	Sleepy (0.751)	Early morning awakening (0.381)	Difficulty sleeping (0.491)
7	Cannot sleep (0.590)	Trazodone (0.649)	Conduct problems (0.425)	Sleeping difficulty (0.709)	Napping (0.362)	Sleep disturbance (0.352)
8	Restless sleep (0.569)	Day-time sleepiness (0.594)	Often awake (0.393)	Less than (num) hours (0.585)	Disturbance in sleep (0.358)	Naps (0.273)
9	Grouchy (0.545)	Melatonin (0.510)	Inattention (0.391)	Interrupted sleep (0.576)	Active (0.316)	Difficulty focusing (0.266)
10	Go to bed (0.540)	Clonidine (0.508)	Naps (0.382)	Difficulty falling asleep (0.549)	Inattention (0.312)	Wakes up at (0.215)

NH, non-Hispanic; Multi, Multiracial.

### 3.3 Racial/ethnic differences in global Peds B-SATED dimensions

Once we unearthed the differences in primary care providers' documentation of Peds B-SATED keywords across racial/ethnic subpopulations using LASSO regression, we sought to understand the odds of patients having the presence of global Peds B-SATED dimensions in their clinical notes. Of patients, 16.2% had one or more sleep behavior keywords present in clinical notes, 37.0% had one or more sleep satisfaction/quality keywords present, 25.2% had one or more alertness/daytime sleepiness keywords present, 13.5% had one or more sleep timing keywords present, 34.4% had one or more sleep efficiency keywords present, and only 2.1% had one or more sleep duration keywords present. Table 2 shows the racial/ethnic differences in global Peds B-SATED dimensions.

**Table 2:** Mixed-effects logistic regression models predicting racial/Ethnic differences in global Peds B-SATED dimensions.

	**Behaviors**	**Satisfaction**	**Alertness**	**Timing**	**Efficiency**	**Duration**
	**aOR**	**aOR**	**aOR**	**aOR**	**aOR**	**aOR**
NH Black	1.02	0.59^**^	1.16^**^	0.89	0.81^**^	0.51
Hispanic	0.94	0.52^**^	0.83^**^	1.88^*^	0.76^**^	2.46
NH multiracial	1.06	0.95	0.98	0.53	1.01	0.30
NH Asian	0.68^**^	0.49^**^	1.04	1.99	0.62^**^	0.31
NH other	1.22	0.75^*^	0.84	3.24^*^	0.82	2.36

Reference = NH white; aOR, adjusted odds ratio; NH, non-Hispanic. Note-level covariates include provider author type and pandemic status. Patient-level covariates include biological sex and age. ^*^p < 0.05. ^**^p < 0.01.

In adjusted models, non-Hispanic Black patients had a lower adjusted odds ratio (aOR = 0.59; 95% CI [0.55, 0.63]; *p* = 0.00) of having the presence of one or more sleep satisfaction keywords, a higher adjusted odds ratio (aOR = 1.16; 95% CI [1.09, 1.24]; *p* = 0.00) of having the presence of one or more alertness/daytime sleepiness keywords, and a lower adjusted odds ratio (aOR = 0.81; 95% CI [0.77, 0.86]; *p* = 0.00) of having the presence of sleep efficiency keywords, compared to non-Hispanic white patients.

In adjusted models, Hispanic patients had a lower adjusted odds ratio (aOR = 0.52; 95% CI [0.48–0.57]; *p* = 0.00) of having the presence of one or more sleep satisfaction keywords, a lower adjusted odds ratio (aOR = 0.83; 95% CI [0.77, 0.90]; *p* = 0.00) of having the presence of one or more alertness/daytime sleepiness keywords, a higher adjusted odds ratio (aOR = 1.88; 95% CI [1.87, 2.99]; *p* = 0.01) of having the presence of sleep timing keywords, and a lower adjusted odds ratio (aOR = 0.76; 95% CI [0.70, 0.82]; *p* = 0.00) of having the presence of sleep efficiency keywords, compared to non-Hispanic white patients.

In adjusted models, non-Hispanic Multiracial patients had a lower adjusted odds ratio (aOR = 0.62; 95% CI [0.54, 0.71]; *p* = 0.00) of having the presence of sleep efficiency keywords, compared to non-Hispanic white patients.

In adjusted models, non-Hispanic Asian patients had a lower adjusted odds ratio (aOR = 0.68; 95% CI [0.56, 0.83]; *p* = 0.00) of having the presence of one or more sleep behavior keywords, a lower adjusted odds ratio (aOR = 0.49; 95% CI [0.42, 0.58]; *p* = 0.00) of having the presence of one or more sleep satisfaction keywords, and a lower adjusted odds ratio (aOR = 0.62; 95% CI [0.54, 0.71]; *p* = 0.00) of having the presence of sleep efficiency keywords, compared to non-Hispanic white patients.

In adjusted models, non-Hispanic Other Race/Ethnicity patients had a lower adjusted odds ratio (aOR = 0.75; 95% CI [0.60, 0.93]; *p* = 0.01) of having the presence of one or more sleep satisfaction keywords and a higher adjusted odds ratio (aOR = 3.24; 95% CI [1.13, 9.27]; *p* = 0.03) of having the presence of sleep timing keywords, compared to non-Hispanic white patients.

During the Covid-19 pandemic, patients had a lower adjusted odds ratio (aOR = 0.86; 95% CI [0.83, 0.90]; *p* = 0.00) of having the presence of alertness keywords, a higher odds ratio of having the presence of satisfaction keywords (aOR = 1.23; 95% CI [1.18, 1.29]; *p* = 0.00), a lower adjusted odds ratio (aOR = 0.79; 95% CI [0.70, 0.88]; *p* = 0.00) of having the presence of timing keywords, a higher odds ratio of having the presence of efficiency keywords (aOR = 1.23; 95% CI [1.18, 1.29]; *p* = 0.00), a lower adjusted odds ratio (aOR = 0.48; 95% CI [0.34, 0.68]; *p* = 0.00) of having the presence of duration keywords, and a higher odds ratio of having the presence of behavior keywords (aOR = 1.37; 95% CI [1.30, 1.45]; *p* = 0.00), compared to prior to the onset of pandemic.

## 4 Discussion

### 4.1 Summary of findings

To our knowledge, this is the first study to assess racial/ethnic differences in NLP training data prior to model training. This study describes LASSO and multilevel modeling approaches that were used to identify these differences in pediatric primary care providers' documentation of Peds B-SATED keywords and global dimensions across racial/ethnic subpopulations. In addition, the most frequently documented Peds B-SATED keyword rankings qualitatively differed across racial/ethnic subpopulations. These racial/ethnic differences of documented Peds B-SATED keywords and global dimensions both extend and align with previous studies of providers' differential documentation of pediatric sleep problems in pediatric primary care settings (Honaker et al., 2018; Carson et al., 2023). Our findings have important implications for future practices in equitably documenting pediatric sleep problems across racial/ethnic subpopulations, which shape how patients experience the clinical workflow from identification in primary care to referral sleep specialty settings in pediatric healthcare institutions.

In addition to our main findings, we found that by using the Peds B-SATED keywords documented in primary care providers' clinical notes, patients' race/ethnicity could be predicted. In addition, the most frequently documented Peds B-SATED keyword rankings qualitatively differed across racial/ethnic subpopulations. These findings indicated that non-Hispanic white patients had keywords that typically noted pediatric sleep problems with more specificity, particularly those that require specialized intervention (Honaker and Saunders, 2018). Yet, among racial/ethnic minoritized patients, keywords commonly included broad descriptions and relevant daytime sequelae of a pediatric sleep problem (e.g., daytime behavior problems, napping, inattentiveness, and irritability). Although these daytime sequelae are necessary for understanding phenotypes of pediatric sleep problems, primary care providers may not have the training to interpret these subclinical characteristics as clinically meaningful proxies for an underlying sleep problem (Golden et al., 2023; Yang et al., 2023). Therefore, it may be a source of concern that these broad descriptions and daytime sequelae are ranked in the top 10 documented keywords for primarily racially/ethnically minoritized patient subpopulations. Future clinical research is needed to further investigate patient–provider factors that may influence these differences in documentation observed across racial/ethnic subpopulations (e.g., patient perception of sleep problems and provider screening practices).

We found that the top three global Peds B-SATED dimensions included satisfaction, efficiency, and alertness/daytime sleepiness (e.g., present in ~25%−37% of patient clinical notes). Although the most commonly monitored in past epidemiologic and population sleep health research, the current study found that only ~2% of patients had the presence of keywords/phrases falling in the sleep duration dimension. We also found that the odds of certain global Peds B-SATED dimensions were both lower and higher in racial/ethnic minoritized subpopulations of patients, compared with non-Hispanic white patients. Thus, efforts to prevent racial/ethnic inequities and manage pediatric sleep problems in primary care require routine and multidimensional screening protocols (Meltzer et al., 2021). In addition, the results highlight that it is also important for future work to consider the heterogeneity in patients' susceptibility (e.g., social and environmental determinants; co-occurring health problems) that can influence their perception of, providers' identification of, pediatric sleep problems (Rubens et al., 2016; Billings et al., 2021; Reynolds et al., 2023).

### 4.2 Ethical implications

The goal of our research was to apply novel and efficient approaches to support the assessment of racial/ethnic differences in NLP training data (e.g., keywords). We approached this study from a “discovery” perspective in our methods, but this study aligns with the future directions for ethical machine learning in pediatric healthcare settings previously outlined in the literature (Huang et al., 2022; Chen et al., 2023). NLP allows pediatric sleep researchers to expand their reach beyond diagnoses and polysomnography data commonly utilized in past healthcare research (Ramgopal et al., 2023). However, these more recent advances that leverage NLP-extracted data are not exempt from racial/ethnic bias that is inherently shaped by historical bias and informed presence (Phelan et al., 2017; Boch et al., 2022). This highlights the important prioritization of innovation and machine learning ethics in pediatric sleep research at the population level (Mhasawade et al., 2021). Leading scholars have recently outlined key ethical and equity-centered processes to consider when using machine learning in healthcare settings (Boch et al., 2022; Walk et al., 2022; Chen et al., 2023). They deemed the process of identifying and addressing biased patterns in data collection, imbalanced or skewed datasets, to be an important step called *preprocessing* (Huang et al., 2022). This preprocessing process typically occurs prior to model training and deployment to prevent biased machine learning models (Huang et al., 2022). Ethical machine learning approaches such as preprocessing are a necessity, not an optional step, to confirm and address any underlying bias in training data sources. This step is particularly important when processing NLP-derived data from clinical documentation and starts at the data collection phase (e.g., clinical protocols for assessment and documentation).

### 4.3 Clinical research implications

Our analyses identified potential differences in clinical documentation of pediatric sleep problems that necessitate future research that examines how primary care shapes the clinical sleep outcomes of racial/ethnic subpopulations. Clinically, these observed differences in our cohort may highlight the importance of clinicians routinely asking about sleep problems in a multidimensional way, which may be a potential pathway for improving the equitable identification of racially/ethnically minoritized patients in pediatric primary care settings (Meltzer et al., 2021). In this pediatric primary care cohort study, we identified differential documentation of Peds B-SATED keywords and global dimensions across racial/ethnic subpopulations, but also a very low presence of various global dimensions in clinical notes overall. For example, the highest prevalence was the presence of the satisfaction dimension at ~37%, while the lowest was the duration dimension at ~2%. These findings allude to the importance of clinical machine learning to rapidly increase the efficiency of enhancing patients' sleep health literacy and education, patient-driven data collection, and the development of clinical decision support tools to aid pediatric primary care providers (Harada et al., 2021; Kang et al., 2021; Ramgopal et al., 2023). Theoretically, efficient clinical workflows for identifying such patients in pediatric primary care can aid research efforts for universal screening of pediatric sleep problems at the population level (Goldstein et al., 2020). Improving the quality and increasing the vastness of data collected in pediatric primary settings can also determine influential social determinants of pediatric sleep problems (Huffstetler et al., 2022). With this level of population-level surveillance in reach using both patient self-report and actigraphy, researchers will be able to develop EHR-embedded machine learning tools for primary care providers to recognize predictors and profiles for distinct pediatric sleep phenotypes (Willis et al., 2022). In the age of precision health, these enhanced clinical workflows and tools are key for improving our ability to equitably reach racial/ethnic subpopulations that may benefit the most from targeted and tailored interventions (Seixas et al., 2020; Honaker et al., 2022).

### 4.4 Limitations

Pediatric sleep problems are multidimensional, but the variation of sleep health definitions and phenotypes among race/ethnicity subpopulations remains limited in pediatrics. Therefore, future work is needed to determine whether the differential patterns observed in providers' documentation of sleep problems are related to unique differences in symptom presentation or literacy across racial/ethnic subpopulations. More specifically, future research should examine whether variation is due to patient self-report or caregiver report, differing community beliefs and literacy about sleep as a health experience, and/or the social and environmental determinants that are potential drivers of existing pediatric sleep disparities (Reynolds et al., 2023; Yang et al., 2023). Using a cross-sectional retrospective cohort design and EHR data, this study is not capable of determining whether provider–patient interactions, implicit cognitive bias of providers or patients, or informed presence influences the ways Peds B-SATED keywords or global dimensions are captured or missed in primary care providers' documentation (Phelan et al., 2017). However, these findings do raise some concerns about differences in Peds B-SATED keyword rankings across racial/ethnic subpopulations. The way a provider documents sleep problems influences how patients' future providers monitor and treat their pediatric sleep problems as clinically meaningful (Honaker and Saunders, 2018). Consequently, varied provider documentation may yield differential care outcomes across racial/ethnic subpopulations of patients. Future studies and replication (e.g., other time periods, clinic settings, and multiple institutions) are needed to understand the factors that cause this differential documentation pattern that we observed across racial/ethnic subpopulations in our pediatric primary care cohort. In addition, the current study included a primarily non-Hispanic Black patient population.

## 5 Conclusion

Overall, the purpose of the study was to assess racial/ethnic differences in providers' documentation of Peds B-SATED keywords and global dimensions. Our findings unearthed racial/ethnic differences in our training data, using both LASSO and multilevel modeling approaches. The three primary results related to racial/ethnic bias in our NLP training data are both informative and addressable. First, we found that primary care providers' documentation of keywords in clinical notes was able to predict patients' race/ethnicity and that this difference was most observable among non-Hispanic Black and non-Hispanic white patient subpopulations. Second, the Peds B-SATED keyword rankings qualitatively differed across racial/ethnic subpopulations. Finally, the results of the mixed-effects models revealed that the presence of global dimensions in clinical notes varied between racially/ethnically minoritized patients compared to non-Hispanic white patients. In the end, the findings confirmed that developing standardized guidelines for documenting pediatric sleep problems in pediatric primary care, in collaboration with specialty sleep providers, may be warranted. This also highlights implications for routine and multidimensional screening in pediatric primary care settings, due to providers' differential patterns of documenting Peds B-SATED keyword and global dimensions that may contribute to differences in clinical outcomes across racial/ethnic subpopulations. In pediatric sleep research, these findings have important implications for identifying a potential sleep care gap that is preventable in pediatric primary care.

## Data availability statement

The data analyzed in this study is subject to the following licenses/restrictions: The datasets presented in this article are not readily available because the data from this study is primarily composed of pediatric electronic healthcare record data that cannot be shared for legal, ethical, and privacy restriction purposes (e.g., patient confidentiality and privacy). Requests to access these datasets should be directed to https://www.nationwidechildrens.org/research.

## Ethics statement

The studies involving humans were approved by the Nationwide Children's Hospital. The studies were conducted in accordance with the local legislation and institutional requirements. Written informed consent for participation was not required from the participants or the participants' legal guardians/next of kin in accordance with the national legislation and institutional requirements.

## Author contributions

MD: Conceptualization, Formal analysis, Funding acquisition, Methodology, Writing—original draft, Writing—review & editing. JS: Conceptualization, Formal analysis, Methodology, Writing—original draft, Writing—review & editing. DC: Conceptualization, Funding acquisition, Supervision, Writing—review & editing.
